# Sexual Dimorphism in Brown Adipose Tissue Activation and White Adipose Tissue Browning

**DOI:** 10.3390/ijms23158250

**Published:** 2022-07-26

**Authors:** Iker Gómez-García, Jenifer Trepiana, Alfredo Fernández-Quintela, Marta Giralt, María P. Portillo

**Affiliations:** 1 Nutrition and Obesity Group, Department of Pharmacy and Food Sciences, Faculty of Pharmacy, University of the Basque Country (UPV/EHU) and Lucio Lascaray Research Institute, 01006 Vitoria-Gasteiz, Spain; ikergomezgarcia98@gmail.com (I.G.-G.); alfredo.fernandez@ehu.eus (A.F.-Q.); mariapuy.portillo@ehu.eus (M.P.P.); 2 BIOARABA Health Research Institute, 01006 Vitoria-Gasteiz, Spain; 3 CIBERobn Physiopathology of Obesity and Nutrition, Institute of Health Carlos III, 01006 Vitoria-Gasteiz, Spain; mgiralt@ub.edu; 4 Department of Biochemistry and Molecular Biomedicine, Faculty of Biology and Institute of Biomedicine at the University of Barcelona (IBUB), Universitat de Barcelona, 08028 Barcelona, Spain

**Keywords:** sexual dimorphism, brown adipose tissue, thermogenesis, browning, sex hormones

## Abstract

The present narrative review gathers the studies reported so far, addressing sex differences in the effects of cold exposure, feeding pattern and age on brown adipose tissue (BAT) thermogenesis and white adipose tissue (WAT) browning. In rodents, when exposed to decreasing temperatures, females activate thermogenesis earlier. Results obtained in humans go in the same line, although they do not provide results as solid as those obtained in rodents. Regarding the effects of overfeeding, interesting sex differences on BAT thermogenic capacity have been reported, and the greater or lower sensitivity of each sex to this dietary situation seems to be dependent on the type of feeding. In the case of energy restriction, females are more sensitive than males. In addition, sex differences have also been observed in thermogenesis changes induced by phenolic compound administration. During sexual development, an increase in BAT mass and BAT activity takes place. This phenomenon is greater in boys than in girls, probably due to its relation to muscle-mass growth. The opposite situation takes place during ageing, a lifespan period where thermogenic capacity declines, this being more acute in men than in women. Finally, the vast majority of the studies have reported a higher susceptibility to developing WAT browning amongst females. The scarcity of results highlights the need for further studies devoted to analysing this issue, in order to provide valuable information for a more personalised approach.

## 1. Introduction

**Figure 1:**
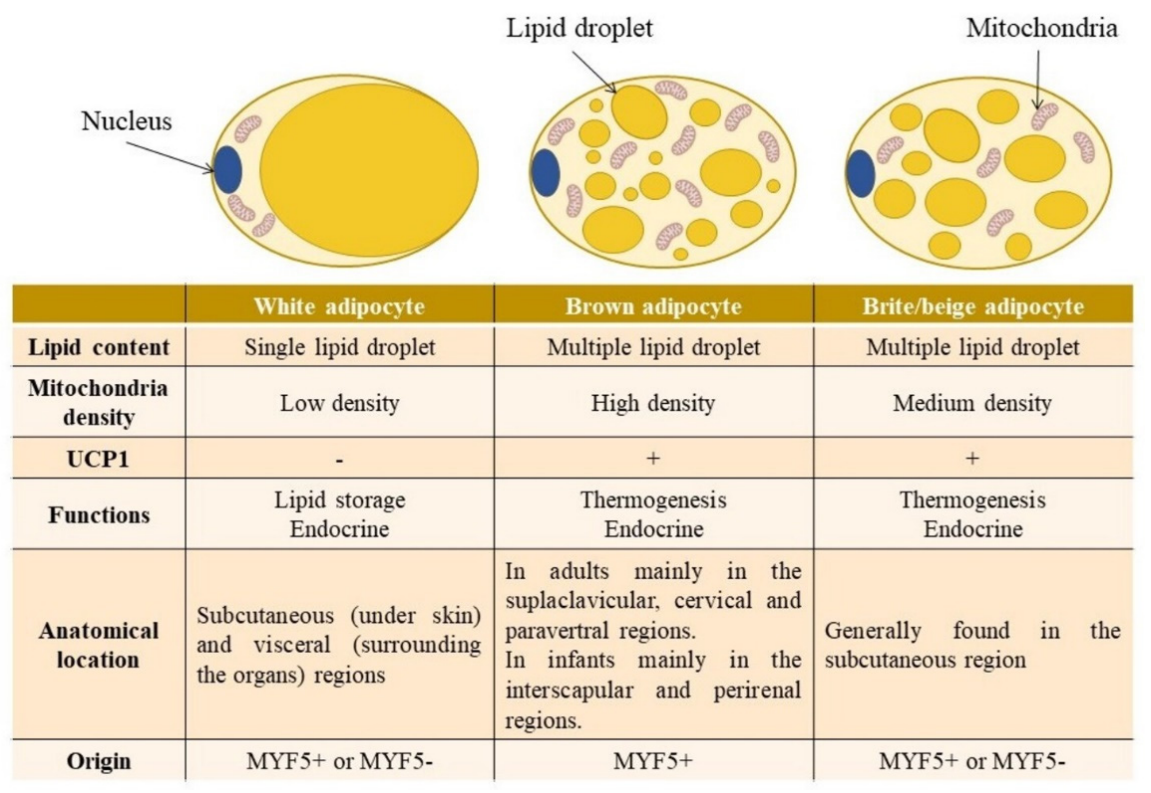
Differences in the metabolic characteristics, functions, anatomical location and origin between white, brown and beige adipocytes. UCP1: uncoupling protein 1; MYF5: myogenic regulatory factor 5.

The main reservoir of fat in the body is white adipose tissue (WAT). White adipocytes, the primary lipid-storing cells, are characterised by having a low mitochondrial density and storing triglycerides in a single lipid droplet [1] (Figure 1). The size of these cells is highly variable among individuals and depends on their fat accumulation. The average size of white adipocytes is reduced in situations of body weight loss and increases in situations of weight gain. Indeed, WAT is available to face situations of food shortage, in which stored triglycerides are mobilized to obtain the energy needed [2]. Several depots of WAT, which show different locations and metabolic features, are found in the body: the subcutaneous adipose tissue, located under the skin, which is the tissue with the highest proportion; and the visceral adipose tissues with intra-abdominal location, which surround the organs, specifically the kidneys, intestines, gonads, heart and the circulatory system [3].

Another type of adipose tissue is brown adipose tissue (BAT), which is characterised by the presence of multilocular adipocytes, which contain many small lipid droplets and a large number of oxidative mitochondria [4] (Figure 1). This fact confers to this tissue its main function, which is the dissipation of energy as heat through mitochondrial respiration, a process known as thermogenesis. This process is possible by virtue of the presence of the uncoupling protein 1 (UCP1) [5].

In addition to the classic white and brown adipocytes, a third type, beige or brite adipocyte, has been discovered. These fat cells appear in WAT, in response to different stimuli such as cold exposure or catecholamine stimulation. This process is termed “browning”. Beige and brown adipocytes have some features in common and some others with WAT. Thus, beige adipocytes express the thermogenic marker UCP1 and present a medium density of mitochondria [4,6,7] (Figure 1). With regard to the gene pattern, beige adipocytes show genes characteristic of WAT, such as adiponectin, adipsin, adipocyte protein 2 gene (*aP2/FABP4*) and peroxisome proliferator activated receptor gamma (*PPARɣ*); along with genes characteristic of BAT, such as *Ucp1*, peroxisome proliferator-activated receptor-gamma coactivator (*Pgc*)*-1a* and cell death-inducing DFFA-like effector A *(Cidea*). Furthermore, beige adipocytes also express T-Box transcription factor 1 (*Tbx1*) and solute carrier family 27 member 1 (*Slc27a1*) genes in a beige-selective manner [8].

Interestingly, beige adipocytes can originate from MYF5+ or MYF5− linage (myogenic regulatory factor 5), depending on the WAT depot. Originally, it was thought that only brown adipocytes could turn into UCP1+ beige adipocytes via MYF5-expressing precursors [9]. However, depending on the WAT fat depot and the stimulation exerted, MYF5+ white adipocytes can undergo browning and acquire UCP1 expression [10]. Nevertheless, the beige adipocytes that develop in the WAT do not share the same provenance as the classic brown adipocytes that emerge before birth [9]. A further origin for beige adipocytes is trans-differentiation, that is, the direct transformation of white adipocytes into beige adipocytes. In situations where energy is demanded, availability or environmental conditions change, where mature adipocytes are able to alter their phenotype from white and trans-differentiate to brown or beige. This process is a two-way process, as trans-differentiation from beige adipocyte to white can also occur [11,12]. 

Finally, during pregnancy and lactation, all three types of adipocytes can trans-differentiate into mammary luminal secretory epithelium cells, defined as “pink adipocyte”, which contribute to milk secretion [13]. Recently, the differentiation of pink to brown adipocytes has also been analysed [14].

WAT, BAT and beige adipocytes are related to several diseases, both directly or by means of the proteins they produce, namely adipokines or batokines [15,16], which not only show autocrine but also paracrine and endocrine functions. It is well known that important differences may exist in risk factors, symptomology, treatment and prognosis of various diseases between men and women. Consequently, further research related to these aspects, which would include and compare both sexes is needed in order to better understand similarities and differences and to be able to apply that knowledge effectively to disease prevention and treatment [17]. Indeed, it is becoming evident that ignoring the influence of sex in research compromises the validity and generalizability of the findings, especially considering that sexual dimorphism takes part in precision nutrition [18].

Sex differences in BAT thermogenesis and WAT browning have been addressed in other reviews. Keuper and Jastroch (2021) have recently published a mini-review focused on sexual dimorphism in innervation, adrenergic receptors, vascularization, and mitochondrial and UCP1 function in human adipose tissue [19]. Pan and Chen (2021) have also reported sex differences in WAT and BAT biology, neuroregulation and secretory function [20]. Moreover, other reviews have analysed the role of sex hormones in BAT metabolism [21]. In order to gather information concerning other aspects of sexual dimorphism in BAT thermogenesis and WAT browning, and thus to complete the reported reviews, the present narrative review focuses on the differences found between both sexes in terms of cold activation, diet activation and age effect, both in preclinical and clinical studies.

## 2. Sexual Dimorphism in BAT Activation Induced by Cold

**Figure 2:**
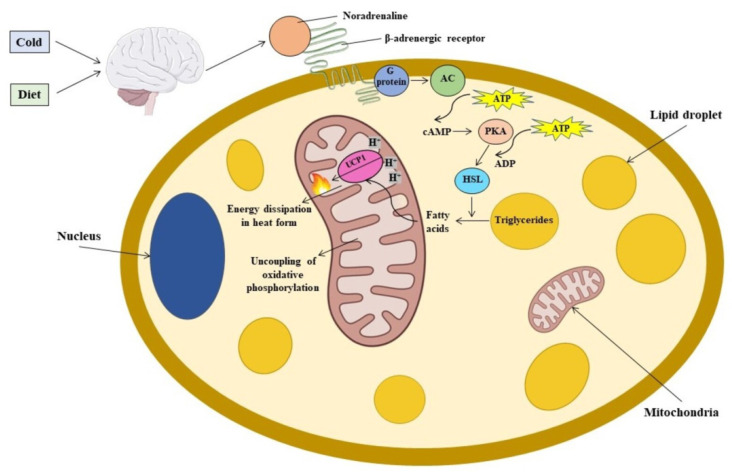
Mechanism of the UCP1 protein activation in BAT. β-adrenergic receptor induces the activation of Gs protein, leading to the production of cyclic adenosine monophosphate (cAMP) by means of adenylate cyclase (AC). This metabolic messenger activates cAMP-dependent protein kinase (PKA), which in turn stimulates hormone-sensitive lipase (HSL), the lipase that acts on diglycerides to produce free fatty acids and monoglycerides. Free fatty acids are then available to be oxidized in mitochondria. ATP, adenosine triphosphate; ADP, adenosine diphosphate; BAT, brown adipose tissue; UCP1, uncoupling protein 1.

Cold exposure is one of the main activators of BAT. When animals are exposed to cold, the transient receptor potential (TRP) channels, present on the body surface, transmit this information to the brain, activating the sympathetic nerves which innervate BAT. Thus, noradrenalin is released, and it binds the β-adrenergic receptors (β-AR) present in BAT adipocytes, triggering a cellular signalling cascade through the second messenger, cyclic adenosine monophosphate (cAMP). This effect prompts the activation of the hormone-sensitive lipase (HSL), one of the main enzymes involved in triglyceride lipolysis. Released fatty acids are oxidised in the mitochondria and activate UCP1 protein, thus triggering thermogenesis [22]. Not only does this signal activate thermogenesis in BAT, but it also stimulates the differentiation of precursor cells to form new brown adipocytes (Figure 2). 

In this section, the results reported in preclinical and clinical studies addressing sexual dimorphism in thermogenesis induced by cold are gathered (Table 1).

### 2.1. Preclinical Studies

Quevedo et al. (1998) reported that acute exposure of male and female rats to low temperature (4 °C for 24 h) led to an increase in UCP1 at the protein level and activity in both sexes, though males showed a higher thermogenic response than females [23]. In contrast, when acclimated to 22 °C, females showed double the amount of UCP1 as males. The authors additionally studied the effect of chronic acclimation from 22 °C to the temperature of thermoneutrality (28 °C), as well as to a lower temperature (18 °C) for seven days. However, at 28 °C, no differences in UCP1 protein were observed between both sexes, female rats showed statistically higher values of UCP1 protein and activity both at 18 °C or at 22 °C. These results suggest a lower temperature threshold for cold-induced thermogenesis in males than in females; BAT in females is activated at 22 °C, whereas male BAT is less sensitive to the cold (thermogenic response around 18 °C). 

The same group conducted a longer study (100 days instead of 7 days) which yielded the same results previously obtained (Roca et al., 1999) for rats exposed to 22 °C (at this temperature thermogenesis starts to be activated in females) [24]. Compared to their male counterparts, ad libitum feeding of chow resulted in higher mitochondrial protein, as well as cytochrome c oxidase (COX) activity and UCP1 in interscapular BAT (iBAT) in female rats, measured by spectophotometry and Western blot, respectively. In addition, gene expression of *Ucp1* and *Ucp2* were also sharply higher in females, suggesting BAT activation induced by cold when kept at 22 °C.

Harshaw et al. (2014) analysed potential sex differences in BAT thermogenesis in C57BL/6 mice pups under environmental conditions where the temperature was cooled from 35.4 °C to 22.5 °C [25]. Interestingly, the females tended to present greater temperature in the interscapular and rump regions than males during the cold phase, which suggests a BAT thermogenesis raise in females. These results are in line with those reported by Quevedo et al. (1998) [23] and (Roca et al., 1999) [24].

Grefhorst et al. (2015) studied two families of paracrine/autocrine hormones or growth factors: bone morphogenetic proteins (BMPs) and fibroblast growth factors (FGFs), which are involved in BAT activity, upon cold exposure [26]. Indeed, BAT expresses several BMP family members, among which BMP7 and BMP8b have been shown to activate BAT directly. Cold exposure induced *Bmp4* and *Bmp8b* expression in BAT, but only *Bmp8b* differed between the sexes. Subsequent to ovariectomy, the expression of this gene was reduced to the levels observed in male mice. This outcome points out that *Bmp8b* expression might be involved in sex-specific differences in BAT activity, and it may also be the result of oestrogen regulation. Concerning the FGF family, BAT *Fgf1*, *Fgf9*, *Fgf18*, and *Fgf21* expression was induced under cold exposure, although only *Fgf1* expression was different between the sexes; males had 2.5-fold higher BAT *Fgf1* than females. 

Following this dimorphic *Bmp8b* expression observed in vivo, the authors performed an in vitro study. When primary brown adipocytes were exposed to diethylstilbestrol (DES), a synthetic agonist of the oestrogen receptors, no changes were observed in *Bmp8b* expression in brown adipocytes. The authors concluded that the effect of DES on BAT *Bmp8b* expression in vivo was likely mediated through an indirect mechanism, presumably via the hypothalamus. Indeed, it has been previously shown that the sex steroid hormone estradiol acts in the central nervous system regulating BAT thermogenesis, specifically in the ventromedial nuclei of the hypothalamus [33].

### 2.2. Human Studies: Child and Adolescents

The number of studies addressing this population is very scarce, and controversial results have been reported. By measuring the skin temperature of the supraclavicular region, by infrared thermography, in 36 adolescents (8.5–11.8 years of age), before and after immersing their hands in moderately cold water for five minutes, boys showed a higher temperature in the BAT area than girls of the same age and body mass index (BMI), both under normal conditions and after cold exposure. This result suggests that boys present higher BAT activity in basal conditions and are more responsive to cold stimulation than girls (Robinson et al., 2019) [27]. In contrast, in a study carried out with 86 children (mean age of 8.5 years) born with normal size for their gestational age, girls showed higher activation of BAT in the supraclavicular region in response to cold exposure (place one hand in cold water at 17–18 °C for five min). Contrarily, this effect was not observed in infants who were born smaller for their gestational age (Malpique et al., 2019) [28]. 

### 2.3. Human Studies: Adults

As early as the 1980s, several questions concerning sexual dimorphism in response to cold stress arose. In his review, Graham (1988) pointed to the significantly limited understanding of this issue [34]. Indeed, several scientists had suggested that gender differences in thermoregulatory responses were plausible due to factors such as differences in body fatness and its distribution, or in body surface area. Certainly, several lines of evidence supported the hypothesis that gender-specific physiological responses to body cooling took place, stating at that time that females were less thermally sensitive to cold water or cold air than males [34]. These pioneering research works were the onset for the scientific community to find adequate answers to the questions related to sexual dimorphism in thermogenic adaptations.

Further studies have confirmed sexual dimorphism in BAT activation in response to cold in adult humans, although these results are not in good accordance with the previous hypothesis. Chen et al. (2013) addressed a study devoted to characterising the relationship between BAT and cold-induced thermogenesis after minimal changes in environmental temperature [29]. Thus, the participants were exposed to 19 °C after staying at 24 °C for 36 h. This intervention allowed the researchers to capture the range of BAT activation rather than its maximal stimulation. Although energy expenditure was higher in women than in men, the relative increase in ^18^F-FDG uptake by PET images was similar in both sexes. Moreover, no significant correlations were found between gender and thermogenesis activation. More recently, in the study addressed by Mengel et al. (2020), 117 adult individuals (mean age 25 years) were exposed to a perfusion of cold water (water was lowered from 32 °C to shivering threshold), and maintained for 120 min. Although a significant decrease in supraclavicular skin temperature was observed in men but not in women, no differences in the thermogenic response were found between sexes (measuring energy expenditure), suggesting no differences in the thermogenesis [30]. In another study carried out with 12 women and 12 men (aged 18–35 years), the authors found that women and men do not differ in relative amount, glucose uptake, and distribution of BAT, but interestingly, they revealed that half the women, but only one man, had potential BAT in dorsocervical adipose tissue, using the PET technique, after cold exposure (16 °C for 5 h) [31]. Recently, a study has been published in which 95 adults (aged 18–50 years) were perfused with low-temperature water (to shivering threshold) in order to activate the BAT. The authors observed that although the difference between the amount of BAT was not significantly different between men and premenopausal women, the activation of thermogenesis was greater in women. This fact was positively correlated with a higher level of oestradiol in blood. It is important to highlight that thermogenesis in women decreased during the menstrual cycle, finding a higher activation of BAT in the follicular phase than in the luteal one, being the thermogenesis in the latter similar to that found in men [32]. Interestingly, the amount of active dorsocervical BAT was also found to be much higher in adult young women than in young men [35]. It must be taken into account that the main BAT depots in neonates are found in the dorsocervical area (as in rodents), and it is thought that these depots gradually disappear with age.

### 2.4. Summary

The results regarding sexual dimorphism in rodents show that thermogenesis starts to be activated in females at higher temperatures than in males (22 °C in females and 18 °C in males). In contrast, at very low temperatures (4 °C) no differences are found between the sexes. In view of these results, it cannot, therefore, be stated that females have a great thermogenic capacity, but rather, that they are more sensitive to cold than males, which leads to an earlier activation of thermogenesis in order to combat this situation. Future in vivo studies should address whether the mechanism of thermogenesis activation is different in both sexes.

As far as human studies are concerned, those focused on children and adolescents are very scarce and inconclusive. In fact, there are just two original manuscripts showing opposite effects. Regarding adults, among the four studies reported, two of them did not show differences between sexes and the other two found higher BAT activity in women, just in one specific area, the dorsocervical. Altogether, the studies addressed in humans do not provide results as solid as those obtained in rodents. The reasons explaining this difference are not clear. A potential explanation may be related to the specific BAT depots analysed. In rodents, the main depot studied is the interscapular BAT which corresponds to the dorsocervical depots in humans. However, the assessment of BAT was performed only in the supraclavicular region in most of the children and young adults. In conclusion, the scarce and controversial data available highlights the need for further research with emphasis on the influence of sex dimorphism in BAT activation in humans at different ages, under cold exposure. 

## 3. Sexual Dimorphism in BAT Activation Induced by Diet

As indicated previously in this review, cold is the main regulator of thermogenesis in BAT through noradrenaline release (Puigsever et al., 1991) [36] . Additionally, thermogenesis can also be activated in response to food intake; namely, diet-induced BAT thermogenesis (DIT) [36]. Though this issue needs further research, several studies in in vivo models point out that diet-induced and cold-induced thermogenesis share the sympathetic nervous system (SNS)-adrenergic receptors (AR) axis [37,38]. Moreover, the vagus nerve is in charge of transmitting the information to the brain in diet-induced thermogenesis [39].

The reported studies dealing with this issue have analysed the effects of overfeeding, using either cafeteria diet or high-fat diets, and those of energy restriction (Table 2). A cafeteria diet provides highly palatable and energy-dense foods to induce an in vivo model of overeating and obesity in rodents, mirroring the modern western food environment. The first studies were carried out approximately 20 years ago. Roca et al. (1999) analysed the potential sexual dimorphism regarding the effects of 100-day cafeteria feeding on BAT thermogenesis [24]. By comparing the control- and the cafeteria-fed groups, the authors observed increased *Ucp1* and *Ucp2* gene expression in males from the latter group. In contrast, this effect was not observed when the comparison was made between control- and cafeteria-diet-fed females, indicating that sex-related differences in thermogenesis response to chronic high caloric diet intake exist. In addition, the gene expression of β3-AR, a key effector of thermogenesis, was reduced both in male and female rats fed with the cafeteria diet, compared with their control counterparts, independently of the sex. In this scenario, so as to study more thoroughly the involvement of SNS-βAR signalling in the gender differences observed in the response to cafeteria diet, in the same study, the authors addressed an in vitro experiment in brown adipocytes. They analysed whether the effect showed in the in vivo model could be reproduced by noradrenaline alone (the main effector of diet effects). They observed that BAT precursor cells from cervical, axillar and interscapular brown fat depots of 4-week-old male mice differentiated and incubated with noradrenaline, similarly to that observed in the in vivo experiment, showed a reduction in the β3-AR gene expression and an increase in *Ucp1* and, at a lesser extent, in *Ucp2* mRNA levels, which followed a dose-dependent pattern. The authors proposed that β-3AR down-regulation occurred as a retroregulation mechanism related to chronic sympathetic activation of BAT.

To study the involvement of sexual hormones in the sex differences observed in response to diet, the same research group carried out another in vitro experiment with primary differentiated brown adipocytes [46]. When the authors incubated adipocytes with testosterone for 24 h, the expression of the α2-AR gene, whose activation has an inhibitory effect on thermogenesis, was up-regulated. Nevertheless, 17β-estradiol, and progesterone at a higher extent, increased β3-AR mRNA levels. In accordance with this, female sex hormones (17β-estradiol, and progesterone) also reduced α2A/β3 adrenergic receptor ratio. These results highlight the key role of sex hormones in adrenergic regulation in BAT. The incubation with noradrenaline prompted a down-regulation of β3-AR gene expression in brown adipocytes co-incubated with sex hormones, in line with the results obtained in their previous study [24]. Therefore, due to the fact that in in vitro experiments the sympathetic nervous control has no influence, it seems that sex hormones play a role in modulating thermogenesis. This fact suggests that sex-dependent differences found in vivo in response to overfeeding might be related to the action of sex hormones.

In view of the results obtained in cultured brown adipocytes, the same group explored the potential gender-related differences in the balance between β3-AR and α2A-AR in BAT, and their relationship with the expression of UCPs, in the course of a 15-day standard or cafeteria-feeding period (Rodríguez et al., 2001). Conversely, in the control rats, thermogenic capacity was higher in females than in males, under cafeteria feeding, in which adrenergic activation occurs, female rats displayed lower thermogenic capacity, in accordance with the results that the group had previously reported in a much longer experiment (100 days) (Roca et al., 1999) [24]. This was due to a lower expression of β3-AR in females fed with a cafeteria diet, which is the most active pathway under noradrenaline stimulation. Furthermore, females also showed a much lower α2A-AR protein expression than males, both in control and in cafeteria-fed rats. Although β-ARs induce stimulatory effects on the thermogenic control and α2A-ARs induce inhibitory effects on this pathway, females displayed a lesser response to overfeeding due to a lower expression of β3-AR, despite lower α2A-AR. This can be explained in terms of β3-ARs becoming more important than other adrenoreceptors under sympathetic nervous system activation (like overfeeding). Therefore, these results suggested that male rats are able to better counteract the effects of overfeeding [40].

Using the same cohort of rats, this research group further reported a reduction in gene expression of *Pparɣ2*, a transcription factor that induces BAT differentiation, and that is a key activator of UCP1 in female rats, but not in males (Rodríguez et al., 2004) [41]. These results suggest that the null activation of thermogenesis in female rats on a cafeteria diet found in the previous study carried out by the group Roca et al. (1999) [24] could be due, not only to the reduction in the expression of β3-adrenergic receptor, but also to the down-regulation of *PPARɣ2*, which prompts UCP1 inhibition.

In other studies, high-fat diets have been used as an obesogenic diet in place of a cafeteria diet. Surprisingly, the results attained are not in accordance with those obtained with a cafeteria diet. In the work reported by Choi et al. (2011), the authors observed that high-fat feeding for eight weeks led to higher body weight at the end of the experimental treatment in males, although not in females. Additionally, female rats showed increased UCP1 protein expression, as well as up-regulation of fatty acid oxidation and higher energy expenditure, compared to males. Regarding sexual hormones, the oestrogen levels were higher in rats fed with the high-fat diet compared to the control group, this increase being much greater in females than in males (30% in females *vs.* 15% in males). This points to a possible role of this hormone in the observed effects. In fact, these authors suggested that high levels of oestrogen in females increased energy expenditure, likely due to an up-regulation of thermogenesis and fatty acid β-oxidation, and inhibition of the lipogenic enzymes [42].

Recently, McCannell et al. (2021) have observed a greater amount of BAT and subcutaneous WAT in males compared to females fed with the same high-fat diet. Nevertheless, in the BAT of female mice under a high-fat diet, a mitochondrial adaptation occurred, consisting of increased Complex I and II respiration, which resulted in higher energy expenditure, this result not being observed in males. The authors suggested that female mice on a high-fat diet have greater metabolic flexibility to adapt to changes that involve higher energy consumption [43]. 

Some studies have analysed the existence of sexual dimorphism concerning the effect of energy restriction on thermogenesis. In the study reported by Valle et al. (2005) female rats fed ad libitum showed a higher increase in UCP1 protein expression and greater energy expenditure than males since oxygen consumption and CO_2_ production grew in the aforementioned group [44]. In contrast, when the animals were submitted to 40% energy restriction for 100 days, females presented a sharp reduction in BAT mass compared with males. In addition, females showed down-regulation of UCP1 protein, an effect that was not observed in males. These results suggest that females decrease energy expenditure under calorie restriction to a greater extent than males, possibly in order to conserve energy and encourage survival.

This cohort of rats was used by the same authors to publish a further study focused on the potential mechanisms involved in the effects described above (Valle et al. (2007) [45]. In this study, restricted females showed reduced content of mitochondrial protein and DNA content, compared with restricted males. In addition, UCP1, as well as lipoprotein lipase (LPL), hormone-sensitive lipase (HSL) and mitochondrial transcription factor A (TFAM) proteins were down-regulated in restricted female rats compared with ad libitum rats, an effect not shown in male groups. Moreover, females fed with the restricted diet showed an increased α2A/β3 adrenergic receptor ratio, which indicates lower thermogenic capacity in females.

### Summary

The preclinical studies described in this section show that the changes induced by the feeding pattern on BAT thermogenesis very much depend on sex. Nevertheless, it is difficult to reach a clear conclusion because only a reduced number of studies have been reported. In addition, concerning the response to overfeeding, controversial results have been published. Although the results observed in rats fed with a cafeteria diet show that only males respond to this dietary pattern with an increase in thermogenic capacity, the results obtained in rodents (rat or mice) fed with a high-fat diet show the opposite, that is, the response induced in females is significantly higher than that of males. Nevertheless, it is important to point out that the studies addressed with cafeteria diet come from the same research group, and were carried out in the same cohort of rats. 

In the case of the high-fat diet, only two studies have been reported, although both reached the same conclusion, that females develop a greater increase in thermogenesis capacity than males. In this scenario, it is not possible to determine whether the differences provided by these studies are a matter of the type of diet, meaning, that either the response of each sex depends on the diet composition, or that they are justified by differences in the experimental designs of the studies. Finally, in the case of energy restriction, only two studies have been reported, and they were both addressed in the same cohort of animals by the same research group. The authors suggested that females, on a restricted diet, showed a lower energy expenditure and lower thermogenic capacity. 

## 4. Sexual Dimorphism in BAT Activation: Effect of Age

As explained in the previous sections, thermogenesis can be modified by exogenous stimuli, such as cold and diet, and these modifications can vary depending on the sex. This led to the question of whether sexual differences can also exist in response to physiological changes, as is the case of age. Several studies have been carried out to give a response to this question (Table 3). Gilsanz et al. (2012) were interested in characterising the changes in BAT that take place during puberty in boys and girls. For this purpose, they examined the prevalence and the volume of BAT at different stages of sexual development in 73 paediatric patients who underwent positron emission tomography (PET)/computed tomography. The authors found a greater mass of BAT, higher activity of this tissue and increased depot size during puberty in boys than in girls [47]. Changes in BAT during sexual development were closely related to gains in muscle volume in both boys and girls. Thus, brown fat and muscle are predicted by pubertal stage and sex. Puberty is characterised by numerous metabolic and hormonal changes, including an increase in the production of growth hormone/growth factors, gonadotropins and sex steroid hormones. Variations in one or more of these factors, known to influence muscle development, could also account for the increase and sex dimorphism in brown fat during adolescence. Finally, BAT is particularly abundant in neonates but few data on sex dimorphism are available. Only a very recent study reports that BAT activity at the posterior cervical region, estimated using infrared thermography, is higher in girls than in boys at age 1 year [48]. 

Thermogenesis changes also take place at the final stages of the lifespan. Thus, the loss of effectiveness in thermogenesis during ageing is well known [53]. Valle et al. (2008) maintained new born female and male rats, under 22 °C for 6, 18 and 24 months [49]. These authors reported that 6 and 18-month-old female rats showed higher UCP1 and COX protein expression and oxygen consumption than males at the same age, prompting higher thermogenic features in female rats. In addition, triiodothyronine (T_3_) levels significantly increased in 6-month-old females compared to male rats. The authors also observed a strong correlation between T_3_ levels and thermogenic activity. Nevertheless, both females and males showed marked BAT atrophy at 24 months of age, due to a reduction in UCP1 and COX protein, and mitochondrial protein.

Results in the same line have been observed in humans. In a trial carried out on 260 individuals, premenopausal women (from 43 to 56 years old) exhibited higher BAT mass and higher activation of this tissue than men. These authors also showed that either the activation or the mass of BAT decreased with age, this effect being more pronounced in men than in women (Pfannenberg et al., 2010) [51]. Yasui et al. (2007) studied the association of both factors, age and sex, with sensitivity to cold in a cohort of 154 healthy men (50 to 84 years; mean 67.3 years) and 180 post-menopausal women (51 to 85 years; mean 66.6 years) [50]. The number of men who had sensitivity to cold, increased with age, with 20.8% of cold-sensitive individuals in the 71–80 years range. However, women whose sensitivity to cold was already high (23.7%) at age 50 to 60 years, did not show any change with aging. In summary, age was significantly associated with sensitivity to cold in men, but not in women. Further, no association between serum hormonal concentrations (estradiol, luteinizing hormone, free testosterone) and sensitivity to cold was found. As declared by the authors, one of the main limitations of this study was that they assessed sensitivity to cold by means of questionnaires, which is not an objective method. Persichetti et al. (2013) also analysed the relationship between age and BAT activity by PET/CT scan in 168 men and 477 women, after exposure to 24 °C for 60 min [52]. The results showed an inverse association between age and BAT mass, and between age and BAT activity. Nevertheless, there were no differences in BAT activity between the sexes. 

### Summary

Two periods of important changes in thermogenesis capacity can be found in lifespan. During sexual development (puberty), an increase in BAT mass and BAT activity takes place. This phenomenon is greater in boys than in girls, probably due to the fact that it is somehow related to the gain in muscle mass. 

The opposite situation takes place during ageing, a lifespan period where thermogenic capacity declines. This decline is stronger in men than in women. Although no studies have been published addressing a potential relationship between this decline and the natural decrease in muscle mass associated with ageing, taking into account the results obtained during puberty, this association cannot be ruled out.

## 5. Sexual Dimorphism in Browning

### 5.1. Preclinical Studies

After adequate stimulation, WAT depots can achieve BAT-like features. In recent years, several studies have suggested that sex differences exist in this white-to-brown adipose tissue process (Table 4). Considering that the innervation of the sympathetic nervous system into WAT is necessary for browning activation [54], Kim et al. (2016) aimed to study the effect of a β3-adrenergic receptor agonist (CL316,243) on browning in male and female C57BL/6 mice [55]. For this purpose, mice were injected intraperitoneally with CL316,243 for β3-adrenergic stimulation. The authors reported that browning induction differed, depending on the WAT location. Thus, UCP1 and PGC-1α protein expression was increased in gonadal WAT (gWAT) of female mice, although this effect was not shown in males. In addition, an up-regulation of cytochrome c oxidase subunit VIIIb (COX8b) protein, a component of the cytochrome c oxidase (enzyme of the mitochondrial electron transport chain) and the tyrosine hydroxylase (TH; enzyme that mediates the rate-limiting step of noradrenaline biosynthesis) was also observed in this tissue in females, along with increased oxygen consumption rate, when compared with males. Furthermore, sex differences were also shown in protein expression of the neurotrophic factor nerve growth factor (NGF) and the brain-derived neurotrophic factor (BDNF), which affect sympathetic innervation, in gWAT of females treated with the β3-adrenergic agonist. By contrast, this sexual dimorphism was not found in inguinal WAT (iWAT), since CL316,243 treatment induced the same TH protein expression in both sexes (no further gene markers were analysed in iWAT). In addition to that, the authors also induced ovarian failure in female mice by injecting intraperitoneally 4-vinylcyclohexene (150 mg/kg for 15 consecutive days) in order to determine whether sex hormones were essential for browning of female gWAT [55]. A significant decrease in both TH and UCP1 protein expression and NGF and BDNF protein expression was observed in gWAT of females.

Seongjoon et al. (2020) studied the role of neuropeptide Y (NPY) on thermogenesis and the potential existence of sex differences [56]. For this purpose, the authors fed NPY−/− or NPY+/+ male and female mice ad libitum until six months of age. UCP1 protein expression in iWAT, a key marker of browning and body temperature, as well as gene expression of *Ucp1*, *Cox7a1*, *Cox8b*, *Pparα*, and *Dio2* were increased in female NPY knock-out mice when compared with wild-type females, but not in male mice. Furthermore, when NPY−/− and NPY+/+ mice were six months old, they received letrozole (0.02 or 0.1 mg/kg in drinking water) for a period of four months in order to inhibit oestrogen production. NPY−/− mediated up-regulation of thermogenic capacity was inhibited in females, as a consequence of *Ucp1*, *Cox7a1*, *Cox8b*, *Pparα*, and *Dio2* gene expression, and UCP1 protein expression. Thus, this data shows that NPY may have a gender-differential effect on fat metabolism and highlight the role of oestradiol in browning regulation. 

Likewise, Miao et al. (2016) also highlighted the key role of oestrogen in the WAT browning process [57]. These authors aimed to study the influence of oestrogen receptor (ER)β on the browning of WAT in mice treated with an ERβ agonist (LY3201; LY) for three days, using old (1-year-old) and young (3-month-old) mice. When 1-year-old female C57BL/6 mice were treated with LY, they appreciated increased gene expression of browning markers *Ucp1*, *Cidea*, *Pparɣ*, *Pgc1β* and *Dio2*, in mammary WAT. No changes in browning markers of subcutaneous WAT were observed in 1-year-old male mice treated with LY. However, when young mice were treated (3-month-old female or male), no changes were showed in UCP1 protein expression, nor in browning-related genes in WAT. In fact, the authors suggested that ERβ is crucial for sympathetic control in aging females. Furthermore, the authors analysed this expression of ERβ in WAT of young and old female mice, and they found that it was higher than in their age-matched male counterparts. In this line, females treated with LY showed a significant increase in both TH protein and β3-adrenoceptor in WAT. Therefore, these results provide evidence that ERβ plays an important role in the induction of the β3-adrenoceptor in WAT, and that this mechanism presents sexual dimorphism in mice. In addition, Zhao et al. (2019) also carried out an in vivo experiment with the aim of analysing whether oestrogen receptors played a role in browning activation in females [58]. When the authors injected tamoxifen intraperitoneally, an oestrogen receptor ligand, to female and male mice for three alternative days, they observed that in the group of females housed under room temperature, UCP1 protein was up-regulated in inguinal WAT and that this effect was not displayed by male mice. Similarly, when these animals were maintained under cold temperatures, UCP1 and cytochrome C proteins were increased in iWAT and gWAT of females treated with tamoxifen, but not in male mice. These results suggest that this oestrogen receptor ligand significantly activates thermogenesis in WAT of female mice.

Other authors have analysed sexual dimorphism in the browning induced by specific types of diets. Servera et al. (2014) fed male and female rats a standard diet, supplemented or not with leucine, during lactation until weaning at 21 days of age [59]. Next, at the age of 6 months, rats were fed a high-fat diet for three additional months. Brite gene markers were increased in iWAT both in males and females. However, in males, gene expression of *Cidea*, *Homeobox C9 (HoxC9)* and *Short stature homeobox (Shox2)* proved to be higher, which would indicate that males better responded to browning induced by this dietary treatment than females. Moreover, early leucine supplementation reduced *Ucp2* gene expression in females fed with the control diet.

Under different experimental conditions, other authors have observed increased browning in females than in males. Lee et al. (2016) used a model of mice fed with a diet deficient in methionine and choline (MCD) for two weeks, a dietary model of liver steatosis [60]. Protein and gene expression of UCP1 and *Elongation of very-long-chain fatty acids-like 3* (*Elovl3)* gene expression were increased in gWAT of female mice fed with the MCD diet, but not in males. The gene expression of *Cox8b*, a key gene in mitochondrial oxidative phosphorylation and a marker of thermoregulation in BAT, was boosted in females on the MCD diet, which could indicate a greater activation of mitochondrial oxidation. On the other hand, these authors also analysed FGF21, a protein that up-regulates browning, acting directly in WAT, as well as intensifying the adrenergic signalling. FGF21 levels were also significantly higher both in the liver and plasma of female mice under the MCD diet, which could be related to the increased browning process in females fed an MCD diet.

Norheim et al. (2019) conducted a study with inbred and recombinant inbred mouse strains fed with a high-fat high-sucrose diet for eight weeks [61]. After that, female mice showed heightened levels of UCP1 expression in gWAT compared to males. To conduct a more in-depth study of the effect of sex hormones, the authors also performed an experiment with gonadectomized C57BL/6J mice fed the obesogenic diet, where they found that Ucp1 staining was higher in females either under chow or high-fat high-sucrose diet, whereas almost no Ucp1 staining was detected in males. These results suggested that gWAT of females is metabolically more active compared to that of males, and that sexual dimorphism in the browning process was regulated by both sex hormones and the obesogenic diet. 

There are also studies where sexual dimorphism was not observed since the changes described were found in both males and females. Thus, Zhuang et al. (2017) focused their study on female and male mice fed with a high-fat diet for ten weeks, with the objective of inducing obesity [62]. Next, the animals were supplemented or not with arachidonic acid for 15 additional weeks, to analyse the effect of this fatty acid on obesity. Mice treated with this fatty acid showed a decreased metabolic activity of iBAT, measured by PET/CT, together with a reduction in the gene expression of browning markers (*Ucp1*, *Prdm16*, *Bmp7*, *Cebpβ*, *Pgc1α*) in iWAT. By contrast, browning markers were augmented in gWAT similarly in male and female mice. 

### 5.2. Human Studies

The activation of browning in a sex-specific manner has also been examined in humans but only using ex vivo studies. Van den Beukel et al. (2015) studied perirenal adipose tissue collected from healthy kidney donors (20 men and 24 women) [66]. In order to observe higher differences in UCP1 expression, these tissue samples were harvested when the outside climate temperature was below 11 °C for one week. In primary cultures obtained from these tissue samples, they found a significant increase in UCP1 protein expression and a tendency to increased PGC-1α protein expression in perirenal tissue of women, though this up-regulation was not shown in men under the same conditions. In addition, adipocytes derived from stem cells of perirenal fat depots also expressed higher levels of UCP1 protein in females than in males.

### 5.3. Summary

Several studies have been carried out to analyse potential sexual dimorphism in WAT browning using different stimuli and experimental situations (treatment with a β3-adrenergic agonist, NPY knocking-out, methionine-deficient diet feeding, arachidonic fatty acid supplementation or high-fat diet feeding), but unfortunately, only one study has been reported using each type of stimulus and thus, comparisons cannot be made. Nevertheless, although obviously, the experimental design in all these studies is very different, with the exception of arachidonic fatty acid supplementation, in the vast majority of the studies females are more prone to developing WAT browning than males. It is noteworthy, that with the exception of the only study in rats by Servera et al. (2014), in all the research pieces these stimuli prompted browning in gonadal WAT of females, instead of iWAT. These results are in accordance with the ex vivo assay performed in human perirenal tissue by Van den Beukel et al. (2015), which showed an up-regulation of the UCP1 marker in women.

With regard to the role of sex hormones in browning sexual dimorphism, the reported studies showed that induction of ovarian failure and inhibition of oestrogen production reduced WAT browning, and that the administration of oestrogen receptor agonists increased reduced WAT browning, in females but no in male mice. Therefore, it can be suggested that oestrogen plays a key role in the activation of the β3-adrenoceptor in WAT inducing browning process in a dimorphic way. Interestingly, this sex hormone regulation is similar to that reported for BAT activation [21].

## 6. The Role of Bioactive Compounds in Browning Sexual Dimorphism

Although a great number of studies have analysed the effect of bioactive compounds on WAT browning [67], to the best of our knowledge, only three recent original articles have been published to address sexual dimorphism regarding this effect (Table 4). Serrano et al. (2018) treated female and male new born mice with resveratrol, orally administered, from day 2 to 20 of life [63]. On day 21, mice were fed a chow diet and then, on day 90, half the animals were assigned to a high-fat diet for 10 additional weeks. Resveratrol treatment was able to significantly increase *Prdm16*, *Pgc-1a*, *Pgc-1b*, *Hoxc9*, *Slc27a1* and *Pparɣ* mRNA levels in iWAT of adult male mice compared with the control group; whereas this effect was not observed in females. The authors concluded that resveratrol supplementation in early life positively benefits male mice in adulthood by up-regulating the browning process. In addition, Asnani–Kishnani et al. (2019) carried out a study with female and male rats fed with a diet supplemented with resveratrol from day 2 after birth to day 20 [64]. In iWAT of male mice supplemented with resveratrol, *Ucp2* gene expression was decreased compared to the control males, whereas this effect was not observed in treated females. However, gene expression of the browning markers *Ucp1*, *Cpt1b*, *Prdm16*, *Tmem26* and *Pgc-1* was not modified in iWAT of male mice supplemented with resveratrol. In addition, the authors performed an in vitro assay with primary cultures of iWAT from those mice. It was observed that the *Ucp1* gene was up-regulated in iWAT primary cultures from resveratrol supplemented males, and down-regulated in treated female mice, compared with their respective controls. These results suggest that resveratrol supplementation modulates browning in a sex-dependent manner, the males being more sensitive to this effect. 

Spina et al. (2019) conducted a study using pterostilbene, a resveratrol derivative [65]. They fed mice of both sexes with a high-fat diet supplemented or not with pterostilbene for 30 weeks, and they observed that, compared to male rats not supplemented with pterostilbene, this phenolic compound tended to increase UCP1 protein expression in iWAT from females, whereas this effect was not showed in males. 

### Summary

The very scarce results reported so far show that there is a sex-dependent response to stilbenes (phenolic compounds) in terms of the browning process. It is important to highlight that there are controversial published data about this issue, which might be due to the different experimental conditions. Therefore, further research is required to fully explore the potential application of polyphenols in browning activation. 

## 7. Concluding Remarks

The studies described in the present review analyse the existence of sexual dimorphism in BAT thermogenesis and WAT browning. In Table 5, a summary of the main conclusions obtained from the studies comprised in the present review is included. An interesting aspect of the review is that it strongly highlights the need for further research on this issue because the published studies bring to light that the changes induced by cold exposure, the feeding pattern or age on BAT thermogenesis depend on sex. Regarding the effect of cold exposure, it seems quite clear that females increase the thermogenic capacity of BAT at higher temperatures than males do, because they are more sensitive to cold, suggesting that females activate thermogenesis earlier than males with the aim of counteracting this situation. Therefore, women display more cold sensation than men. As far as the effects of feeding are concerned, future studies should address the role of diet composition in this sexual dimorphism, since the reported results surprisingly show that whereas male rodents are more prone to cafeteria diet-induced increase of BAT thermogenic capacity, female rodents are more sensitive to the effects of high-fat feeding. With regard to adolescence, in this age group, the thermogenic capacity sharply increases in boys, suggesting that this effect might be due to the higher muscle mass expansion in this gender. However, the opposite result was reported during ageing. In addition, although it seems that females are more prone to develop browning, in the vast majority of the cases, controversial results have been reported in gonadal WAT depots. Thus, this review highlights that sex differences should be taken into account in future studies of thermogenic adipose tissue in humans.

Finally, it is important to highlight, that the original manuscripts compiled in this review show several limitations. On the one hand, the studies carried out in humans under cold conditions do not show solid results compared to that of rodents, which might be due to the fact that BAT depots analysed in animals do not correspond to that studied in humans. On the other hand, in the case of articles published addressing sex differences in the thermogenic capacity across the lifespan, more studies analysing the relationship between the thermogenesis declination along the life and muscle mass loss are needed. Moreover, the information reported about the sexual dimorphism in rodents under several dietary interventions, or the effects on WAT browning is still rather scarce; therefore, further research is needed in order to increase the knowledge in this field. 

## Figures and Tables

**Table 1:** Sexual dimorphism in thermogenesis induced by cold in preclinical and clinical studies.

Author	Animal Model	Experimental Model	Effects	Mechanism of Actions
**Preclinical Studies**				
Quevedo et al. (1998) [23]	Male and female Wistar rats	Acute exposure (4 °C for 24 h) to rats previously acclimated to 22 °C Chronic acclimation from 22 °C to 28 °C or 18 °C for 7 days	Higher increase in thermogenic capacity in males than in females At 22 °C and 18 °C higher thermogenic capacity in females than in males At 28 °C no differences between sexes	↑ *Ucp1* gene expression ↑ UCP1 protein levels ↑ UCP1 protein levels ↑ activity (GDP binding to mitochondria)
Roca et al. (1999) [24]	Male and female Wistar rats	Exposure at 22 °C for 100 days	Higher BAT thermogenic capacity in females than in males	↑ Mitochondrial proteins ↑ COX activity and UCP1 ↑ *Ucp1* and *Ucp2* gene expression
Harshaw et al. (2014) [25]	Male and female C57BL/6 mice pups	Cooling temperature from 35.4 °C to 22.5 °C	Greater temperature in the interscapular and rump regions in females than in males	Not explained
Grefhorst et al. (2015) [26]	Male and female C57Bl/6J mice	Exposure at 23 °C or 4 °C for 24 h	__	2.5-fold higher BAT *Fgf1* in males than in females 35-fold lower BAT *Bmp8b* in males than in females
**Clinical studies in children and adolescents**				
Robinson et al. (2019) [27]	36 adolescents (16 boys and 20 girls) Age: 8.5–11.8	Hand immersion in a moderately cold water for 5 min.	Higher temperature in the BAT area in boys than in girls, measured by infrared thermography	Not explained
Malpique et al. (2019) [28]	Infants: 39 boys and 47 girls Age: 8.5 years ± 0.1 years BMI: 17.5 ± 0.4 kg/m^2^, distributed by born small-for-gestational age (SGA) or appropriate-for-gestational age (AGA)	Hand immersion in a moderately cold water (17–18 °C) for 5 min.	In AGA: ↑ BAT activity higher in girls than in boys, measured by infrared thermography. In SGA: no differences between sexes	Not explained
**Clinical studies in adults**				
Chen et al. (2013) [29]	14 men and 10 women Age: 28.1 ± 7.3 years BMI: 20.0–27.0 kg/m^2^	Exposure at 19 °C after staying at 24 °C for 36 h	Higher energy expenditure in women than in men No differences in BAT activity measured by ^18^F-FDG PET/CT	Not explained
Mengel et al. (2020) [30]	58 men and 59 women Age: 25.1 ± 3.6 years BMI: 22.3 ± 1.7 kg/m^2^	Perfusion of cold water (lowered from 32 °C to shivering threshold) for 120 min.	↓ Supraclavicular skin temperature only in men, measured by thermosensors in iBAT skin. No differences between sexes in energy expenditure	↑ T3 levels in men
Fletcher et al. (2020) [31]	12 men and 12 women Age: 18–35 years BMI: 18.5–25.0 kg/m^2^	Cold exposure (16 °C) for 5 h	No differences between women and men, with the exception of: ↑ BAT activity in dorsocervical BAT in women *vs.* men, measured by ^18^F-FDG uptake (PET)	Not explained
Herz et al. (2021) [32]	95 adults Age: 18–50 years BMI: 20–55 kg/m^2^	Perfusion of cold water (to shivering threshold) for 60 min	↑ Thermogenesis in women *vs.* men ↓ Thermogenesis during the menstrual cycle	↑ oestradiol

^18^F-FDG PET/CT: 18F-fluorodeoxyglucose positron emission tomography/computed tomography; AGA: appropriate-for-gestational age; BAT: brown adipose tissue; BMI: body mass index; BMP: bone morphogenetic protein; BMP8b: bone morphogenetic protein 8b; COX: cytochrome c oxidase; FGF: fibroblast growth factor; FGF1: fibroblast growth factor 1; GDP: guanosine diphosphate; SGA: small-for-gestational age; T3: triiodothyronine; UCP1: uncoupling protein 1.

**Table 2:** Sexual dimorphism in thermogenesis induced by diet in preclinical studies.

Author	Animal Model	Experimental Model (Diet)	Effects	Mechanism of Actions
**Overfeeding**				
Roca et al. (1999) [24]	Male and female Wistar rats	Ad libitum feeding with control diet or cafeteria diet for 100 days	Cafeteria diet: Higher BAT thermogenic capacity in males than in females	Cafeteria diet: ↑ *Ucp1* and *Ucp2* gene expression
Rodríguez et al. (2001) [40] Rodriguez et al. (2004) [41]	Male and female Wistar rats	Ad libitum feeding with control diet or cafeteria diet for 15 days	Control diet: Higher BAT thermogenic capacity in females than in males Cafeteria diet: Higher BAT thermogenesis in males than in females	Control diet: ↑ UCP1 protein expression Cafeteria diet: ↓ β3 AR protein expression. ↓ α2A-AR protein expression ↑ Gene expression of *Pparɣ2*
Choi et al. (2011) [42]	Male and female Sprague–Dawley rats	Ad libitum feeding with control diet or high-fat diet for 8 weeks.	High-fat diet: ↑ Body weight only in males Females *vs.* males: ↑ Fatty acid β-oxidation ↑ Energy expenditure	↑ UCP1 protein expression ↑ Oestrogens
McCannell et al. (2021) [43]	Male and female C57BL6/N mice	Ad libitum feeding with control diet or high.fat diet for 10 weeks	High fat diet: ↑ BAT mass ↑ sWAT in males High fat diet, females *vs.* males: ↑ Energy expenditure	↑ Complex I and II respiration
**Energy restriction**				
Valle et al. (2005) [44] Valle et al. (2007) [45]	Male and female Wistar rats	Ad libitum feeding or restricted feeding (60% of ad libitum intake) for 100 days.	Ad libitum feeding: ↑ Energy expenditure in females *vs.* males Restricted diet: Females *vs.* males ↓ Energy expenditure ↓ BAT mass.	↑ UCP1 protein expression ↓ UCP1 protein expression ↓ Mitochondrial protein. ↓ Mitochondial DNA content ↓ UCP1, LPL, HSL and TFAM protein expression. ↑ α2A/β3 adrenergic receptor ratio

BAT: brown adipose tissue; HSL: hormone-sensitive lipase; LPL: lipoprotein lipase; NGF: nerve growth factor; *PPARɣ:* peroxisome proliferator activated receptor gamma; sWAT: subcutaneous white adipose tissue; TFAM: transcription factor A, mitochondrial; UCP1: uncoupling protein 1; UCP2: uncoupling protein 2.

**Table 3:** Sexual dimorphism in the effect of age on BAT thermogenesis in preclinical and clinical studies.

Author	Animal Model	Experimental Model	Effects	Mechanism of Actions
**Preclinical Studies**				
Valle et al. (2008) [49]	Male and female Wistar rats	Ad libitum feeding maintained under 22 °C and sacrificed at 6, 18 and 24 months of age.	Higher thermogenesis in female than in male	Females *vs.* males: ↑ UCP1 and COX protein expression ↑ T3 levels
**Clinical studies in children and adolescents**				
Gilsanz et al. (2012) [47]	Pediatric patients: 38 males and 35 females Age: 4–19.9 years BMI: 25.4–106.4 kg/m^2^	BAT presence and activity was measured by ^18^F-FDG PET/CT.	Lower thermogenesis and BAT depot size during puberty in female than in male	Not explained
**Clinical studies in adults**				
Yasui et al. (2007) [50]	154 healthy men Age: 50 to 84 years BMI: 20.7–24.1 kg/m^2^ and 180 post-menopausal women Age: 51 to 85 years BMI: 23.0–23.8 kg/m^2^	Questionnaire-based allotted to “Sensitive to cold” group.	Age was significantly associated with sensitivity to cold only in men	Not explained
Pfannenberg et al. (2010) [51]	124 men and 136 women Age: 11–82 years BMI: 15.5–40.8 kg/m^2^	BAT mass and activity was measured by ^18^F-FDG PET/CT in thermoneutral conditions	Higher BAT thermogenesis in premenopausal women (43–56 years-old) than in men	Not explained
Persichetti et al. (2013) [52]	168 men and 477 women Mean age: 56.25 ± 15.96 years BMI: 25.23 ± 64.73 kg/m^2^	BAT presence and activity was measured by ^18^F-FDG PET/CT scan at 24 °C	Inverse trend between age and BAT mass; and between age and BAT activity	Not explained

^18^F-FDG PET/CT: 18F-fluorodeoxyglucose positron emission tomography/computed tomography; BAT: brown adipose tissue; BMI: body Mass Index; COX: cytochrome c oxidase; T3: triiodothyronine; UCP1: uncoupling protein 1.

**Table 4:** Sexual dimorphism in WAT browning induced by different stimuli in preclinical studies.

Author	Animal Model	Experimental Model (Diet)	Effects	Mechanism of Actions
**Preclinical Studies (Miscelanea)**				
Kim et al. (2016) [55]	Female and male C57BL/6 mice.	Daily intraperitoneally administration of CL316,243 (a β3-adrenergic receptor agonist; 1 mg/kg d) for 5 days. Intraperitoneal injection of 4-vinylcyclohexene (150 mg/kg) for 15 consecutive days in order to create ovarian failure.	Females *vs.* males: ↑ Browning of gWAT.	↑ UCP1 and PGC-1α protein expression ↑COX8 protein expression ↑ TH protein expression ↑ NGF and BDNF protein expression ↓TH and UCP1protein expression ↓NGF and BDNF protein expression
Seongjoon et al. (2020) [56]	Male and female NPY−/− and NPY+/+ mice	NPY knock-out Letrozole administration (0.02 or 0.1 mg/kg in drinking water) for 4 months to inhibit oestrogen production	Females *vs.* males: ↑ Thermogenenic capacity in iWAT ↓ Thermogenenic capacity in iWAT	↑ UCP1 protein expression ↑*Ucp1*, *Cox7a1*, *Cox8b*, *Pparα,* and *Dio2* gene expression ↓ UCP1 protein expression ↓*Ucp1*, *Cox7a1*, *Cox8b*, *Pparα*, and *Dio2* gene expression
Miao et al. (2016) [57]	Female and male C57BL/6 mice 3-month-old 1-year-old	Mice treated with LY3201 a ERβ agonist, (0.04 mg/d) for 3 days.	One-year-old females *vs.* males ↑ Browning in mammary WAT. Three-month-old females *vs.* males No differences between sexes	↑ ERβ (immunochemistry) ↑ TH and β3-adrenoceptor (immunochemistry).
Zhao et al. (2019) [58]	Female and male C57BL/6 mice	Intraperitoneal injection of tamoxifen, an oestrogen receptor ligand, (25 mg/kg/d) for 3 alternative days. Cold temperature and cold exposure	Room temperature: Females *vs.* males: ↓ igWAT and gWAT weight in females. ↑ Browning markers in igWAT Cold exposure: Females *vs.* males: ↑ Browning markers in igWAT and gWAT	↑ UCP1 protein expression in iWAT ↑ UCP1 and cytochrome C proteins in iWAT and gWAT
**Preclinical studies (dietary treatments)**				
Servera et al. (2014) [59]	Female and male rats	Standard diet supplemented or not with leucine (2%) while lactation until weaning at 21 days of age. Then, at the age of 6 months high- fat diet or control diet for 3 additional months.	Males *vs.* females: ↑ Browning capacity	↑ *Cidea*, *Hoxc9* and *Shox2* gene expression.
Lee et al. (2016) [60]	Female and male C57BL/6 Mice	Diet deficient in methionine and choline (MCD) for 2 weeks	Females *vs.* males: ↑Activation of mitochondrial oxidation. ↑ Browning in gWAT.	↑ UCP1 protein expression ↑ *Cox8b*, *Ucp1*, and *Elovl3* gene expression ↑ COX8b protein expression ↑ FGF21 protein expression in liver ↑ FGF21 in plasma
Norheim et al. (2019) [61]	Female and male inbred and recombinant inbred mouse	High-fat high-sucrose diet for 8 weeks	Females *vs.* males ↑ Browning of gWAT.	↑ UCP1 protein expression
Zhuang et al. (2017) [62]	Female and male C57BL/6J mice	High-fat diet for 10 weeks, and arachidonic acid (AA; 10g/kg) for 15 additional weeks.	No differences between sexes	
**Preclinical studies (bioactive compounds).**				
Serrano et al. (2018) [63]	NMRI female and male mice	RSV (2 mg/kg) supplementation from day 2 to 20 (orally). On day 90, half the animals were assigned to a high-fat diet for 10 additional weeks.	Males *vs.* females ↑ Browning of iWAT.	↑ *Prdm16*, *Pgc-1a*, *Pgc-1b*, *Hoxc9*, *Slc27a1* and *Pparɣ* gene expression.
Asnani-Kishnani et al. (2019) [64]	NMRI female and male mice Primary cultures of iWAT from mice treated with RSV	RSV included in a standard diet 2 mg/kg body weight/day) for 18 days.	Males *vs.* females ↓ Browning markers in iWAT.	↓ *Ucp2* gene expression in males. ↑ *Ucp1* gene expression in iWAT adipocytes from males ↓ *Ucp1* gene expression in iWAT adipocytes from females
La Spina et al. (2019) [65]	Female and male C57BL/6 mice	PT included in a high-fat diet (352 mol/kg body weight/day) for 30 weeks.	Females *vs.* males ↑ Browing in iWAT	↑ UCP1 protein expression

AA: arachidonic acid; BDNF: brain-derived neurotrophic factor; BMP7: bone morphogenetic protein 7; CIDEA: cell death activator; COX7a1: cytochrome c oxidase subunit 7a1; COX8b: cytochrome c oxidase subunit 8b; DIO2: Iodothyronine deiodinase 2; ELOVL3: ELOVL fatty acid elongase 3; FGF21: fibroblast growth factor 21; gWAT: gonadal white adipose tissue; HOXC9: homeobox C9; iBAT: interscapular brown adipose tissue; igWAT: inguinal WAT; iWAT: interscapular white adipose tissue; MCD: diet deficient in methionine and choline; NGF: nerve growth factor; NPY: orexigenic hormone neuropeptide Y; *PPARα*: peroxisome proliferator activated receptor alpha; PT: pterostilbene; RSV: resveratrol; SHOX2: short stature homeobox 2; TH: Tyrosine Hydroxylase; UCP1: uncoupling protein 1; UCP2: uncoupling protein 2.

**Table 5:** Summary of the main sex differences observed in the reported studies.

**Cold Influence**		
Preclinical studies	Females *vs.* males: ↑ Thermogenic capacity [23,24,25,26]	Both in humans and animals, females seem to be more sensitive to cold
Clinical studies (adolescents)	Controversial [27,28]	
Clinical studies (adults)	Women *vs.* men: ↑ Thermogenic capacity [29,30,31,32]	
**Diet Influence**		
Cafeteria diet	Females *vs.* males: ↓ Thermogenic capacity [24,40,41]	Thermogenic capacity induction could depend on the dietary intervention
High-fat diet	Females *vs.* males: ↑ Thermogenic capacity [42,43]	
Energy restriction	Females *vs.* males: ↓ Thermogenic capacity [44,45]	
**Age Influence**		
Preclinical studies	Females *vs.* males: ↑ Thermogenic capacity [49]	With the exception of adolescents, females seem to be more prone to activate thermogenesis than males
Clinical studies (adolescents)	Boys *vs.* girls: ↑ Thermogenic capacity [47]	
Clinical studies (adults)	Women *vs.* men: ↑ Thermogenic capacity [50,51,52]	
